# Random versus Deterministic Descent in RNA Energy Landscape Analysis

**DOI:** 10.1155/2016/9654921

**Published:** 2016-03-02

**Authors:** Luke Day, Ouala Abdelhadi Ep Souki, Andreas A. Albrecht, Kathleen Steinhöfel

**Affiliations:** ^1^Informatics Department, King's College London, London WC2R 2LS, UK; ^2^School of Science and Technology, Middlesex University, London NW4 4BT, UK

## Abstract

Identifying sets of metastable conformations is a major research topic in RNA energy landscape analysis, and recently several methods have been proposed for finding local minima in landscapes spawned by RNA secondary structures. An important and time-critical component of such methods is steepest, or gradient, descent in attraction basins of local minima. We analyse the speed-up achievable by randomised descent in attraction basins in the context of large sample sets where the size has an order of magnitude in the region of ~10^6^. While the gain for each individual sample might be marginal, the overall run-time improvement can be significant. Moreover, for the two nongradient methods we analysed for partial energy landscapes induced by ten different RNA sequences, we obtained that the number of observed local minima is on average larger by 7.3% and 3.5%, respectively. The run-time improvement is approximately 16.6% and 6.8% on average over the ten partial energy landscapes. For the large sample size we selected for descent procedures, the coverage of local minima is very high up to energy values of the region where the samples were randomly selected from the partial energy landscapes; that is, the difference to the total set of local minima is mainly due to the upper area of the energy landscapes.

## 1. Introduction

There is a great diversity in recent research on RNA secondary structure predictions, including refinements of well-established methods such as Mfold [1] and RNAfold [2], kinetic folding simulations, modelling of cotranscriptional folding, and sampling techniques focussing on approximations of the partition function over all secondary structures or specifically for metastable conformations. We briefly recall various aspects of RNA folding simulation and energy landscape analysis, in particular those that inspired the work presented in this paper.

Flamm and Hofacker provide an overview of methods for kinetic folding simulations in [3]; see also the detailed summary by Schuster [4]. While basic kinetic moves are addition and deletion of single base pairs, Flamm et al. [5] introduced the shift move, which is a combination of a base pair removal and a base pair addition where one position remains invariant. The shift move aims at the simulation of “defect diffusion” reported in [6], which tries to capture the process where the position of a bulge in a helix may move along a helix as a result of rapid base pair formation and dissociation.

Cotranscriptional folding is generally acknowledged as describing the process of how RNA folding happens* in vivo* [7]. As pointed out in [3, 8], RNA is transcribed at a rate of only ≈ 30–40 nucleotides per second, where the nascent chain starts folding as soon as it leaves the ribosome. Since helices formed by the incomplete chain may be too stable to refold later on, cotranscriptional folding may drive the folding process to a well-defined folded state that is different from a minimum free energy conformation. In a recent experimental study, Solomatin et al. [9] argue in favour of multiple RNA folding pathways to different biologically active conformations (where the authors include the wider perspective of protein folding).

RNA energy landscape analysis in the context of metastable conformations is presented, for example, in [10–15]. The Barriers tool [10] processes the output of the RNAsubopt program by Wuchty et al. [16] and returns all metastable conformations located in an energy range Δ*E* above the minimum free energy conformation, along with a variety of additional information about the distribution of local minima. We utilise RNAsubopt plus Barriers for generating the information about local minima in partial energy landscapes, which includes recording the run-time. However, the run-time is not compared to descent methods, since the reduction of RNAsubopt to the essential steps of generating the set of local minima would certainly be faster than the recorded times for RNAsubopt plus Barriers execution. Modifying RNAsubopt for such a task, where indeed the sets of local minima are identical to the current RNAsubopt plus Barriers results, is beyond the scope of the present paper.

Lorenz and Clote introduce in [14] the RNAlocopt tool for sampling and approximating the total number of metastable conformations using the partition function. However, currently the RNAlocopt tool has only been implemented by using the Turner 1999 energy model without dangling ends.

Li and Zhang [13] focus on the computation of the set of all possible locally optimal stack configurations over the ensemble of putative stacks, where a new heuristic procedure is utilised for the pathway analysis between local minima. The method targets conformations within a predefined energy range above the minimum free energy conformation and the authors expect the method to be applicable to sequences of up to 250 nt. Saffarian et al. [15] consider the generation of all locally optimal secondary structures assembled from a set of thermodynamically stable helices. The computational experiments for six sequences of length up to 405 nt indicate a relatively short run-time. Huang et al. [11] propose a helix-based heuristic for capturing at least significant subsets of local minima of an RNA folding space. Helices are classified by five loop types that are closed by a given helix. The construction of folding pathways utilises dynamic programming that ensures the correct nesting and juxtaposition of structural elements, where a number *k* of best candidates is considered at each step of the construction of a folding pathway (breadth first search). For fixed values of *k*, the run-time is estimated by *O*(*k* ^2^ *n* ^3^) energy function evaluations. Kucharík et al. [12] introduce a new connectivity model of attraction basins within energy landscapes, along with the new tool RNAlocmin that is designed for generating sets of local minima based upon modified Boltzmann sampling and steepest descent within RNA energy landscapes. The authors present various comparisons to RNAlocopt [14] regarding the coverage of local minima within a given time frame, which turn out to be in favour of RNAlocmin, partly with large differences in the number of detected local minima.

While RNAlocmin is already relatively fast, we are looking in the present paper at run-time improvements by randomising the descent within attraction basins. Furthermore, we are interested in the coverage of local minima by deterministic and random descent methods. We note that, by using randomised strategies, the completion of steepest descent is not further guaranteed. For large samples even a moderate time improvement of the descent procedure for each individual sample can result in a significant speed-up of the overall processing time. In the present paper, we take RNAlocmin [12] as deterministic steepest descent benchmark method for comparison. The implementations utilised in the present paper are accessible via http://kks.inf.kcl.ac.uk/projects/RandomDescent.php.

## 2. Materials and Methods

### 2.1. RNA Structure

In formal terms, a nested secondary structure of an RNA sequence of length *n* is a node-labelled, undirected graph *G* = (*V*, *E*), where *V* = {1,…, *n*}, *E*⊆*V* × *V*, and *L*(*V*) = {*A*, *C*, *G*, *U*}, such that

(1) (*i*, *j*) ∈ *E*⇔(*j*, *i*) ∈ *E*;
(2) ∀*i*  (*i* ∈ {1,…, *n* − 1}→(*i*, *i* + 1) ∈ *E*) (backbone bonds);
(3) for 1 ≤ *i* ≤ *n*, there exists* at most* one *j* ≠ *i*, *i* ± 1, such that (*i*, *j*) ∈ *E*, where *L*(*i*) and *L*(*j*) comply with Watson-Crick pairs or *G*–*U* (*U*–*G*);
(4) 1 ≤ *i* < *k* < *j* ≤ *n*, (*i*, *j*) ∈ *E*, and (*k*, *ℓ*) ∈ *E* imply *i* ≤ *ℓ* ≤ *j*.

### 2.2. RNA Folding Landscapes

The energy landscape of an RNA sequence *R*, denoted by *L*(*R*) = [*C*, *N*, *E*], can be described by three components: a set of secondary structure conformations *C*, a neighbourhood function *N*, and a free energy evaluation function *E*. The conformation space *C* consists of secondary structures as defined above and computed by tools such as RNAsubopt [2]. It is important to distinguish between two types of conformation spaces: noncanonical and more restricted canonical spaces. A conformation is canonical, if for every base pairing (*i*, *j*) there exists another base pairing (*i*′, *j*′) adjacent to (*i*, *j*) at position (*i* + 1, *j* − 1) and/or (*i* − 1, *j* + 1). In noncanonical conformations, isolated base pairs are admitted. Here, we consider canonical conformation spaces only.

The neighbourhood function *N*_*S*_ of a secondary structure *S* defines the adjacency of the conformation space *C*. For the secondary structure *S*, its neighbourhood *N*_*S*_ is a set of structures that are reachable from *S* by applying a single operation from a move set, *S* → *S*′ ∈ *N*_*S*_. Flamm et al. [5] describe two move sets for RNA folding, a basic move set consisting of insertion and deletion of base pairs and a move set where a shift move to facilitate chain sliding is included. In the present work, we consider the insertion and deletion move set, with the reason being that the Barriers implementation of the two move sets generates shift moves only for noncanonical structures. The basic move set is therefore defined in the following way:

(1) Single or double insertion:
  (a) A single base pair may be inserted at position (*i*, *j*), if an existing helix is extended; that is, (*i* + 1, *j* − 1) and/or (*i* − 1, *j* + 1) are paired.
  (b) Two base pairings may be inserted at positions (*i*, *j*) and (*i* + 1, *j* − 1), if *i* or *j* is not adjacent to an existing base pair belonging to the same helix; that is, *i* − 1 and *i* + 2 or *j* + 1 and *j* − 2 are unpaired.
(2) Single or double deletion:
  (a) A single base pairing (*i*, *j*) may be deleted, if its removal does not result in a noncanonical structure.
  (b) Two base pairings (*i*, *j*) and (*i* + 1, *j* − 1) may be deleted,
    (i) if positions *i* − 1 and *i* + 2 are unpaired,
    (ii) if position *i* − 1 is the closing base of a different helix and *i* + 2 is unpaired,
    (iii) if *j* + 1 is unpaired and *j* − 2 is unpaired,
    (iv) if *j* + 1 is the opening base of a different helix and *j* − 2 is unpaired.

Additionally, the moves must also satisfy the secondary structure rules as described above, including a minimum length of hairpins of 3. The number of possible neighbours is bounded by *O*(*n* ^2^), where *n* is the length of the structure. The implementation RNAbor for studying statistics of RNA structural neighbours has been introduced in [17]. RNAbor computes the number and Boltzmann probabilities of all structures having base pair distance *d* to an input structure *S*. RNAbor uses dynamic programming and has a complexity of *O*(*n* ^4^). Currently, RNAbor works for noncanonical neighbour spaces and uses an older version of the nearest neighbour energy model.

The energy function *E* : *C* → *ℝ* calculates the free energy of secondary structures and can be calculated by using, for example, the RNAeval tool from the Vienna RNA Package [2]. Finally, a structure *S*_*m*_ ∈ *C* is metastable (or a local minimum) of the landscape if all its neighbours have higher or equal energy; that is, ∀*S*  (*S* ∈ *N*_*S*_*m*__ → *E*(*S*) ≥ *E*(*S*_*m*_)).

### 2.3. Main Features of RNAlocmin

Here, we briefly describe the main features of RNAlocmin as presented in [12]. The RNAlocmin tool [12] from the Vienna RNA Group accepts as input a set {*S*} of RNA secondary structure conformations and calculates for each structure *S* its corresponding local minimum conformation that defines the attraction basin to which *S* belongs. The underlying method implemented by RNAlocmin is a descent algorithm. RNAlocmin implements three types of descent: (1) a gradient or steepest descent, (2) a first-lower descent, and (3) a random first-lower descent. Along with the local minima structures *S*_*m*_ and their free energies *E*(*S*_*m*_), RNAlocmin counts the total number *c*({*S*}, *S*_*m*_) of input structures *S* that fold into each particular local minimum *S*_*m*_. As the number of input structures is typically much larger than the number of local minima, some local minima must be reached by multiple input structures. The values *c*({*S*}, *S*_*m*_) therefore provide some insight into the number of structures belonging to attraction basins of the energy landscapes, and consequently the potential size of those basins.

The input conformations are converted into a numerical representation, where for each base pairing (*i*, *j*) the opening position *i* is stored at its closing position *j* and the closing position is stored at its opening position. All unpaired positions are set to 0. For example, the structure .(((...))).. of length 12 is represented numerically by

**Table:** 

*i*	1	2	3	4	5	6	7	8	9	10	11	12
Structure	·	(	(	(	·	·	·	)	)	)	·	·
*S*[*i*]	0	10	9	8	0	0	0	4	3	2	0	0

**Figure 1:**
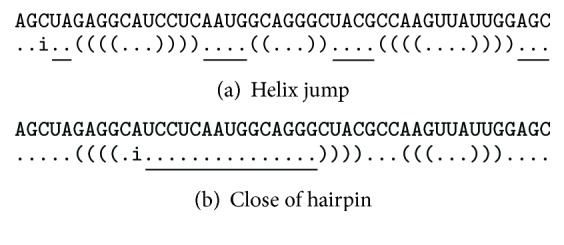
Search for valid base pair (*i*, *j*) positions. (a) By using the numerical representation of secondary structure, it is possible to jump over helices in the search for valid *j* positions. (b) If searching within a hairpin of a helix, then the search can be terminated once a closing bracket is found.

The numerical representation supports the efficient search for potential closing positions *j* of an unpaired open position *i*.  Figure 1 illustrates typical scenarios for finding a suitable *j*-position, given position *i*: (a) the search starts from *j* = *i* + 1. If *S*[*j*] > *j*, then *j* is the first position of a helix and *j* is updated to *S*[*j*] + 1. For example, as for the structure .(((...))).., if *i* = 1 and *j* = 2, then *S*[2] = 10 and *j* updated to 11; (b) position *j* is the closing of a base pairing within a hairpin region where *S*[*j*] < *j*. As indicated in the figure, insertion checks only positions where a potential pairing is possible according to the current structure. In the first case (a), a base pair cannot be inserted between *i* + 1 and *S*[*j*] for a number of values *j*; that is, the search for a suitable *j* “jumps over helices.” The second case (b) occurs if *i* is within the hairpin region of a helix, which is recognised from *S*[*j*] < *j*.

Like for the Barriers tool, it is possible to generate canonical local minima by using RNAlocmin through enabling an optional no-loose-pairs (-noLP) parameter. Also like Barriers, if the -noLP parameter is enabled, then shift moves are not generated. It is important to note that the canonical neighbourhood generated by RNAlocmin differs slightly from that generated by Barriers. The neighbourhood generated by RNAlocmin is larger than the Barriers neighbourhood, because it admits double insertion or deletion of base pairings, if both *i* and *j* are adjacent to a pairing. More specifically, the RNAlocmin implementation of the double insertion move considers both potential inner and outer pairings. For example, if positions (*i*, *j*), (*i* − 1, *j* + 1), and (*i* + 1, *j* − 1) of the structure ..((.i..((...)).j.)).. can form valid pairings, then RNAlocmin evaluates both possibilities:

 
(1)  ..(((i..((...)).j)))..
 
(2)  ..((.i(.((...)))j.))..

However, the outer double insertion move (1) is not valid according to the basic move set rules defined above, and it is not generated by Barriers. Considering both inner and outer double insertion results in some neighbours being evaluated twice. Additionally, RNAlocmin admits double deletion where both *i* and *j* are adjacent to a pairing that will not be removed; for example, ..((i(...)j)).. → ..((.......)).. is generated by RNAlocmin, but not by Barriers.

For a sample *M* of input secondary structures, the time complexity to calculate local minima by using RNAlocmin is *O*(*M* × *kn* ^2^ *E*_*n*_), where *E*_*n*_ is the complexity of energy evaluation and *k* is the maximum number of descent steps to a local minimum. RNAlocmin offers two choices for energy evaluation: energy_of_structure() and energy_of_move(). The energy of the structure method energy_of_structure() is equivalent to calling the RNAeval tool with time complexity *E*_*n*_ = *O*(*n*). The energy of the move method energy_of_move() is a local energy update procedure that was introduced in version 2.1.0 of the Vienna RNA Package and has time complexity *E*_*n*_ = *O*(1) [12].

### 2.4. Descent Procedures

Here, we describe three descent algorithms implemented by RNAlocmin and their modification that make them compatible to the canonical local minima produced by the Barriers tool. In particular, the insertion and deletion move functions implemented by RNAlocmin were changed according to the move set described in Section 2.2.

#### 2.4.1. Gradient Descent

The gradient or steepest descent algorithm calculates and evaluates on each iteration the free energy of* all* neighbouring conformations reachable from some structure *S* by insertion or deletion of base pairs. The input conformation is firstly evaluated using the energy_of_structure() function, and then a search for neighbouring moves is performed.

If a position *i* is unpaired, then a search is conducted for valid closing positions, such that (*i*, *j*) satisfies the move set conditions described previously in Sections 2.2 and 2.3. When a valid pairing position is found, then its energy is evaluated by using energy_of_move(). If the energy returned is lower than all previously seen structures, then the structure is remembered. If a position *i* is paired, then the pairing (*i*, *S*[*i*]) is deleted in case it does not violate the move set conditions. Each iteration continues from the lowest found free energy structure, or steepest neighbour, until a local minimum is found.

#### 2.4.2. First-Lower Descent

First-lower descent simplifies the gradient descent by searching for the first energy improvement. The neighbours of the current secondary structure are evaluated by starting from position *i* = 1 of the current secondary structure (see also Figure 1) until a lower energy neighbour is found. Consequently, whenever a lower energy neighbour is found, the search restarts from position *i* = 1 of the lower energy neighbour until a local minimum is found.

#### 2.4.3. Random First-Lower Descent

In RNAlocmin, random first-lower descent works by, on each iteration, generating and storing all neighbour transition moves according to the RNAlocmin description in Section 2.3; that is, all potential (*i*, *j*) pairing or deletion positions are stored.

Instead of generating all moves, randomly shuffling, and then evaluating until a lower move found, as it is done in RNAlocmin, the new random descent works by starting the search for a move from a random position on the secondary structure. Whenever a valid move is found its energy is evaluated.

The list of moves is then randomly shuffled and the shuffled list of moves is evaluated until a lower energy move is found. If no move from the list results in lower energy, then a local minimum has been found. However, this random first-lower descent is implemented only for noncanonical structures within the RNAlocmin framework. We implemented a modified random first-lower descent procedure for dealing with canonical structures. The new random descent works by starting the search from a random position, *i*, of the current structure. Whenever a lower energy move is found between *i* and *j* ≥ *i* + 1, the structure is updated with the move and the search restarts from another random position *i* in the updated structure. If no lower energy neighbour is found, then the search restarts from position *i* = 1, which means the current structure is tested for being a local minimum.

### 2.5. RNA Sequences

Ten 3′ untranslated region (UTR) sequences were identified such that their lengths allow for adequate generation of partial energy folding landscapes.  Table 1 provides information on the ten human 3′UTR sequences identified from the NCBI and Ensembl databases.

The partial energy landscape of each sequence was generated by using the Vienna RNA Package tools RNAsubopt (version 2.1.7) and Barriers (version 1.5.2).  Table 2 shows the total number of canonical structures, |*C*_*δE*_|, and local minima, *ν*, generated by using RNAsubopt and Barriers within an energy offset *δE* of the MFE conformation.

The energy offsets of partial landscapes were chosen in such a way that the total number of conformations generated by RNAsubopt is between 11 × 10^6^ and 16 × 10^6^. For example, a comparable number of ~15 × 10^6^ conformations are the output generated by RNAsubopt for five instances: GMEB1, LIG3, HTR3E, HLA-G, and ALDH4A1. However, the ratio of local minima in the conformation space |*C*_*δE*_|/*ν* is, for example, for HLA-G = 17.4 and for GMEB1 = 34.0; that is, GMEB1 has over twice the number of local minima for a comparable number of considered low energy conformations. The ratio |*C*_*δE*_|/*ν* (see last column in Table 2) affects the selection of *M*, since, for comparable values of |*C*_*δE*_|, the lower the ratio is, the more the samples are required to cover the larger number of local minima. In more detail, the settings we used with regard to RNAsubopt and Barriers [2] are as shown in Algorithm 1.

## 3. Results

We compare the performance of the three descent algorithms in terms of run-time performance and number of observed local minima.

In order to evaluate the three descent procedures, *M* initial canonical conformations were randomly selected from the top quarter of the energy-sorted partial landscapes, with a subsequent calculation of local minima by using the modified RNAlocmin (version 1.0) tool. Structures that are randomly selected from the highest energy region allow us to sample conformations belonging to a multitude of basins within the partial energy landscape. The initial structures are extracted by using the Linux command “tail -n num 〈RNAsubopt_Input〉 Output”. Then, a random number is generated and the structures are extracted from the top quarter region.  Table 3 shows the percentage of local minima found by each descent method for a random set of conformations in comparison to the number of local minima returned by Barriers.

Over all ten cases, gradient descent results in the smallest number of observed local minima, except for three cases: CBR1, ALDH4A1, and AQP5. For these three cases random descent folds into a slightly smaller number of local minima with a maximum difference compared to gradient of 4.12% for ALDH4A1. This difference suggests that at least for a small number of conformations random descent in folding simulations can take a different folding pathway to a different, possibly lower energy, local minimum compared to gradient descent. First-lower descent displays the largest number of local minima. The overall average difference is 3.89% for first-lower and 1.89% for random first-lower compared to gradient descent. We note that for a given *M* selection the results of random first-lower descent differ only marginally (coverage of local minima as well as run-time), which is why only a single representative run is displayed in Table 3.

**Figure 2:**
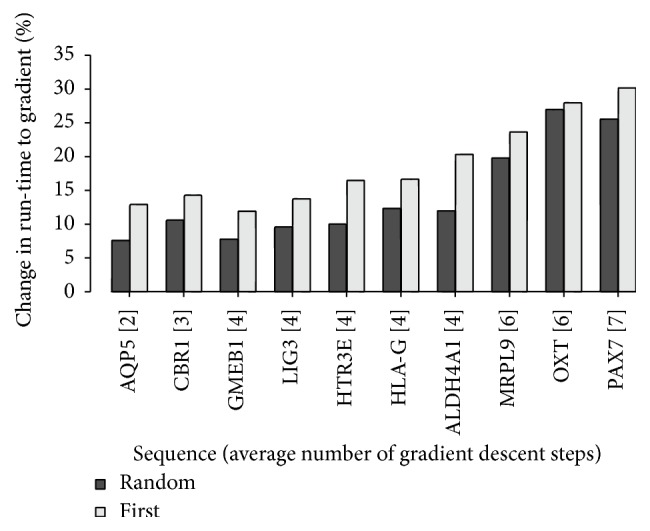
Descent steps: speed-up in % relative to gradient descent in run-time of first-lower and random first-lower for *M* = 500.

Figure 2 shows the percentage of run-time improvement of random first-lower and first-lower compared to gradient descent for the small number of *M* = 500 (% as speed-up relative to gradient descent).  Table 4 reports the corresponding average number of gradient descent iterations over the *M* samples pathways to local minima. For sequences, such as AQP5, where the improvement in run-time is relatively small, the average number of descent iterations is also small. For shorter sequences, such as PAX7, the average number of iterations is larger. Thus, for our dataset, the run-time improvement suggests a correlation to the number of gradient descent iterations: for the largest number of random first-lower descent iterations (= 7, 8, 9) the average speed-up in percentages (= 24.19%) is more than twice as large compared to the corresponding average value calculated for the lowest numbers of iterations (= 4, 5).

Since the energy offsets in Table 2 were chosen in such a way that instances have a comparable number of conformations within their respective partial energy landscapes, a larger subset of the complete energy landscape is considered for shorter sequences. An underlying principle of energy-driven RNA folding is that base pairings stabilise conformations. A secondary structure is said to be saturated, if it is not possible to insert a base pairing without violating the rules of secondary structures. As a larger portion of the full energy landscape is generated for shorter sequences, the top quarter of the energy-sorted partial landscape will consist of a larger number of unsaturated conformations. The comparison of descent methods for saturated structures is unlikely to lead to any considerable differences in the run-time, because the cost of deleting base pairings is equal for each descent method. However, for unsaturated structures the more time-expensive insertion operations are required for the folding process into local minima.

**Figure 3:**
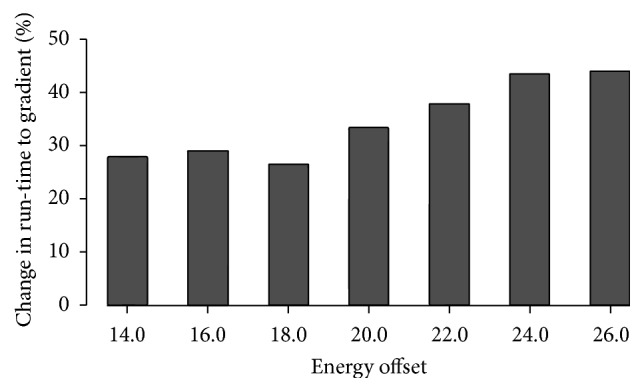
Increasing energy offset: percentage change in run-time of random descent compared to gradient for OXT and *M* = 3 × 10^6^ (speed-up in % relative to gradient descent).

The run-time correlation to descent iterations suggests that random first-lower and first-lower descent are likely to perform particularly well for unsaturated structures.  Figure 3 shows the run-time difference in percentages for random first-lower descent compared to gradient descent for increasing values of energy offsets for a particular sequence. We note that for this analysis the *M* samples were randomly selected from the unsorted partial conformation space as returned by RNAsubopt; see also Table 5 for absolute values.

The reason for the selection of *M* samples from the entire partial space, instead from the highest energy region, is due to the large number of conformations. For example, the number of conformations for offset 26.0 in Figure 3 is just over 3 billion; see Table 5. The sorting procedure implemented in RNAsubopt is memory-expensive, and therefore offsets resulting in very large numbers of conformations exceed the standard desktop computer memory range. In general, a significant run-time improvement is likely to be achieved when folding process proceeds from higher energy conformations within the partial energy landscape.

As can be seen from Table 3, first-lower descent and random first-lower descent detect on average for the datasets considered more local minima compared to gradient descent (57.43% and 55.43% compared to 53.54%). Moreover, for all ten partial energy landscapes and the selected values of *M*, either first-lower descent or random first-lower descent detects more local minima than gradient descent. On the other hand, the run-time is shorter on average and, except for OXT with *M* = 10^6^, on all sequences, see Table 6.

**Figure 4:**
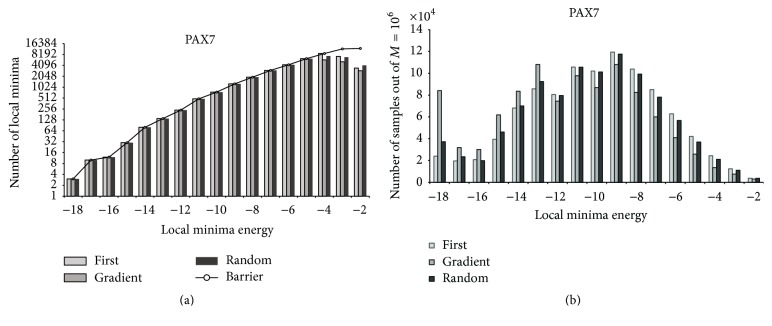
Coverage of Barriers local minima by the three descent methods.

Figure 4 displays the coverage of local minima by different descent methods for PAX7 with *M* = 10^6^. The energy values of local minima are rounded to integer values. As can be seen from the upper part, the coverage complies with the Barriers data for low energy values up until −4 kcal/mol. The differences in higher values are clearly the result of the random selection of *M* sample structures. The lower part of Figure 4 provides information about the distribution of sample structures within attraction basins. The left hand side indicates the number of samples (out of *M*) “attracted” by local minima of a certain energy value. The figure shows that gradient descent is steering many samples into low energy local minima, whereas first-lower descent and random first-lower descent cover a wider range of local minima.

**Figure 5:**
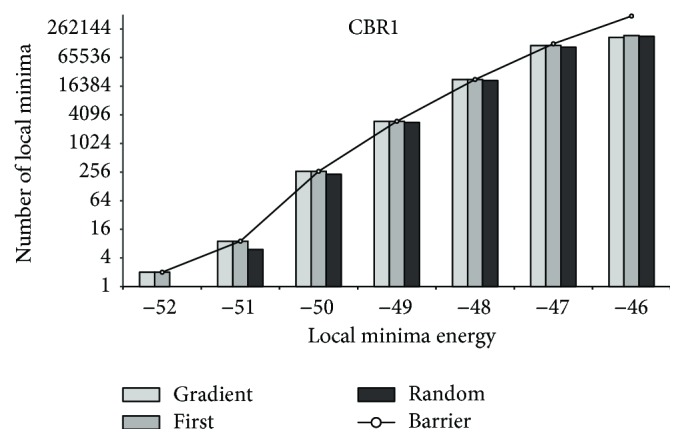
CBR1: local minima coverage.

**Figure 6 fig6:**
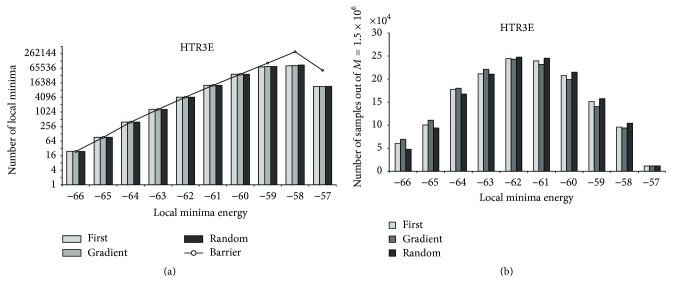
HTR3E: local minima coverage.

**Figure 7:**
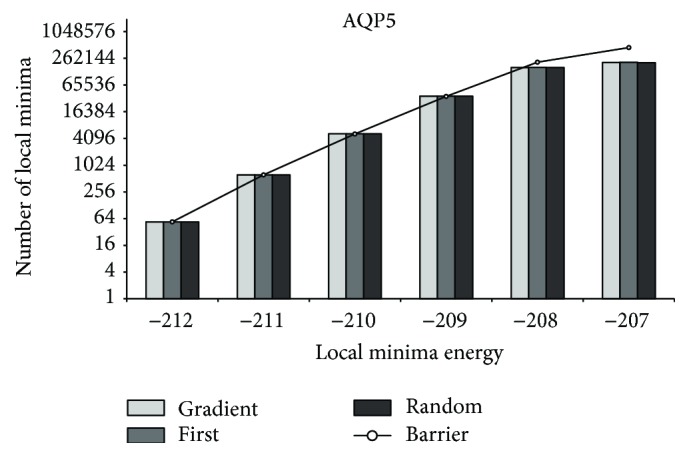
AQP5: local minima coverage.

Figures 5 [Fig fig6]–7 demonstrate that similar distributions of observed local minima are valid for partial energy landscapes induced by sequences of varying lengths (for CBR1, random first-lower descent does not find local minima of lowest energy for the runs we executed). For HTR3E, we observe similar distributions of local minima as for PAX7. Thus, the “main loss” of local minima appears to happen at the high energy range. Consequently, one could argue that for sufficiently large *M* the decent methods return a high coverage of local minima, if the *M* samples are randomly selected outside the region of interest.

## 4. Conclusion

A fundamental principle of structural biology is that sequence encodes structure and in turn structure provides insights into function. However, the rate at which RNA structures are being determined experimentally lags significantly behind that of proteins. Elucidating the structures of RNA conformations presents many experimental and computational challenges. Computational RNA secondary structure prediction and analysis are most commonly based on thermodynamic stability where the focus is on the single minimum free energy conformation. However, it is now commonly acknowledged that* in vivo* RNAs may not always fold into their minimum free energy conformations and may instead fold into an ensemble of structural states. Consequently, this suggests that the information flow for RNAs is better described by sequence →* folding landscape *→ structure → function.

In this work, we applied three descent methods to partial RNA energy landscapes and compared run-time and coverage of local minima on random sample sets of conformations taken from the partial energy landscapes induced by ten RNA sequences. In comparison to gradient descent, we obtained on average a total run-time improvement of about 16.6% along with an increase of 7.3% in observed local minima for first-lower descent and a shorter run-time of 6.8% on average with 3.5% more observed local minima for random first-lower descent. One of our main observations is that for all three descent procedures the coverage of local minima produced by Barriers is very high for energy values close to the minimum free energy structure and up until the region where the samples are randomly selected within the partial energy landscapes. We reiterate that, in principle, for sufficiently large *M*, the decent methods return a high coverage of local minima, if the *M* samples are randomly selected outside the region of interest. However, it would be difficult to make an* a posteriori* assessment of the exact coverage of local minima within the region of interest, which also extends to the* a priori* selection of the sample size *M*.

Here, we focused primarily on the run-time and coverage of local minima for a sample of randomly selected conformations. In future work, we aim to analyse statistical properties of deterministic and random descent folding pathways to local minima. It is an open question if, for highly unstable conformations, a deterministic first-lower descent will, in most cases, converge to the same folding pathway taken by randomised descent. For three of the cases we considered, the number of observed local minima is slightly smaller for random descent in comparison to gradient descent. If folding starts from highly unstable states, then the question is how strongly differs the local minima ensemble produced by random descent in terms of structural features, free energy, and energy barriers when compared to deterministic descent.

## Figures and Tables

**Algorithm 1:**
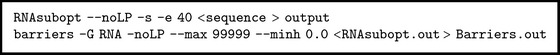


**Table 1:** 3′UTR sequences; *ℓ* denotes the length of sequences (number of nucleotides).

Number	Gene name	*ℓ*	NCBI reference number	Transcript ID
1	PAX7	99	NM_002584.2	ENST00000375375
2	OXT	99	NM_000915.3	ENST00000217386
3	GMEB1	113	NM_024482.2	ENST00000361872
4	LIG3	124	NM_002311.4	ENST00000262327
5	CBR1	284	NM_001757.2	ENST00000290349
6	HTR3E	302	NM_001256614.1	ENST00000360323
7	HLA-G	386	NM_002127.5	ENST00000360323
8	ALDH4A1	400	NM_170726.2	ENST00000290597
9	MRPL9	407	NM_031420.2	ENST00000368830
10	AQP5	504	NM_001651.3	ENST00000293599

**Table 2:** Partial energy landscapes; *ℓ* denotes the length of sequences (number of nucleotides), *δE* is the energy offset above the MFE structure, |*C*_*δE*_| is the number of secondary structures within the partial energy landscape defined by *δE*, and *ν* is the number of local minima within *C*_*δE*_ identified by R NAsubopt and Barriers.

Number	Gene name	*ℓ*	*δE*	|*C*_*δE*_|	*ν*	|*C*_*δE*_ | /*ν*
1	PAX7	99	16.2	14,340,878	50,861	282.0
2	OXT	99	15.0	14,164,430	74,426	190.3
3	GMEB1	113	10.5	15,845,050	466,093	34.0
4	LIG3	124	13.0	15,525,022	317,284	48.9
5	CBR1	284	6.0	10,987,435	643,999	17.1
6	HTR3E	302	9.0	15,095,701	533,316	28.3
7	HLA-G	386	4.2	15,791,146	906,393	17.4
8	ALDH4A1	400	5.4	15,186,200	540,609	28.1
9	MRPL9	407	6.2	14,023,048	41,979	334.0
10	AQP5	504	5.5	11,173,352	714,812	15.6

**Table 3:** Observed local minima: percentage of observed local minima, *ν*_ob_, relative to *ν* found by each descent procedure for the same set of *M* conformations (i.e., last three columns in %); *ℓ* equals the length of sequences and *ν* is the number of local minima identified by R NAsubopt and Barriers within the partial energy landscapes.

Number	Gene	*ℓ*	*ν*	*M* × 10^6^	Gradient	Random	First
					lm(*M*)	lm(*M*)	lm(*M*)
1	PAX7	99	50,861	1.0	63.55	72.64	74.53
2	OXT	99	74,426	1.0	61.74	67.41	72.70
3	GMEB1	113	466,093	2.0	54.54	56.54	57.76
4	LIG3	124	317,284	1.0	42.72	45.50	49.12
5	CBR1	284	643,999	1.5	49.40	48.95	52.07
6	HTR3E	302	533,316	1.5	42.42	44.34	43.55
7	HLA-G	386	906,393	2.5	51.49	52.92	52.99
8	ALDH4A1	400	540,609	1.5	51.06	46.94	51.69
9	MRPL9	407	41,979	1.0	60.44	61.08	61.19
10	AQP5	504	714,812	2.0	58.02	57.98	58.72

Average					53.54	55.43	57.43

**Table 4:** Descent iterations: average number of iterations for *M* = 500 and speed-up (in %) in random first-lower descent run-time compared to gradient.

Gene	Gradient	Random	% change
AQP5	2	4	7.69
CBR1	3	5	10.66
GMEB1	4	6	7.86
LIG3	4	6	9.67
HLA-G	4	5	12.39
HTR3E	4	5	10.11
ALDH4A1	4	6	12.08
MRPL9	6	7	19.88
OXT	6	8	27.04
PAX7	7	9	25.65

**Table 5:** Increasing energy offset: number of observed local minima and run-time (minutes) for increasing energy offset for gene OXT. Note: *M* = 3 × 10^6^ randomly sampled from the full partial landscape.

*δE*	|*C*_*δE*_ | × 10^6^	Gradient time	Random time	First time	Gradient lm(*M*)	Random lm(*M*)	First lm(*M*)
14.0	7.3	14.80	10.65	11.60	45,656	46,005	48,138
16.0	26.7	15.47	10.98	11.48	65,923	70,003	74,939
18.0	86.4	15.83	11.61	14.55	87,307	96,911	106,807
20.0	248.7	17.27	11.47	14.85	108,334	124,566	141,783
22.0	638.5	19.08	11.82	11.78	125,422	150,296	176,904
24.0	1,466.5	21.61	12.17	12.80	138,447	173,275	210,951
26.0	3,018.7	22.28	12.48	12.20	147,261	191,784	241,460

**Table 6:** Run-time in minutes of R NAsubo pt, Barriers , and modified RNAlocmin descent procedures for *M* conformations. Note: RNAlocmin energy evaluation using energy_of_move().

Number	*ℓ*	*M* × 10^6^	RNAsubopt time	Barriers time	Gradient time	Random time	First time
1	99	1.0	1.48	6.43	5.42	4.03	3.40
2	99	1.0	1.40	5.78	3.03	3.67	3.62
3	113	2.0	1.48	13.63	3.95	6.68	3.38
4	124	1.0	1.70	10.52	3.62	3.27	3.12
5	284	1.5	5.87	18.52	7.22	6.45	6.18
6	302	1.5	6.58	33.90	7.12	6.04	5.93
7	386	2.5	9.25	60.12	12.67	11.10	10.57
8	400	1.5	10.25	53.30	6.62	5.82	5.27
9	407	1.0	15.12	40.12	5.18	4.15	3.95
10	504	2.0	15.75	47.63	9.10	8.40	7.92

Total			68.88	289.84	63.94	59.61	53.34
